# The Wnt/β-catenin signaling pathway in the tumor microenvironment of hepatocellular carcinoma

**DOI:** 10.20892/j.issn.2095-3941.2021.0306

**Published:** 2021-10-12

**Authors:** Kaiting Wang, Xinyao Qiu, Yan Zhao, Hongyang Wang, Lei Chen

**Affiliations:** 1 School of Life Sciences, Fudan University, Shanghai 200438, China; 2 Fudan University Shanghai Cancer Center, Department of Oncology, Shanghai Medical College, Fudan University, Shanghai 200032, China; 3 Institute of Metabolism & Integrative Biology (IMIB), Fudan University, Shanghai 200438, China; 4 The International Cooperation Laboratory on Signal Transduction, Eastern Hepatobiliary Surgery Hospital, Second Military Medical University, Shanghai 200438, China

**Keywords:** Wnt/β-catenin signaling, HCC, tumor microenvironment, immunotherapy

## Abstract

The Wnt/β-catenin signaling pathway regulates many aspects of tumor biology, and many studies have focused on the role of this signaling pathway in tumor cells. However, it is now clear that tumor development and metastasis depend on the two-way interaction between cancer cells and their environment, thereby forming a tumor microenvironment (TME). In this review, we discuss how Wnt/β-catenin signaling regulates cross-interactions among different components of the TME, including immune cells, stem cells, tumor vasculature, and noncellular components of the TME in hepatocellular carcinoma. We also investigate their preclinical and clinical insights for primary liver cancer intervention, and explore the significance of using Wnt/β-catenin mutations as a biomarker to predict resistance in immunotherapy.

## Introduction

Globally, primary liver cancer has been upgraded to the third leading cause of cancer-related mortality according to the latest data released by International Agency for Research on Cancer. Hepatocellular carcinoma (HCC) is the most frequent primary liver cancer, accounting for about 70%–85%^1^. In HCC, most patients diagnosed at early or intermediate stages can potentially be cured by surgery, including liver resection, transplantation, and ablation or palliative locoregional therapies such as transarterial chemoembolization. However, the disease recurrence rate is high. Approximately 40% of patients diagnosed with advanced disease can be palliatively treated with systemic therapy when curative therapies and transarterial chemoembolization are no longer available^2^.

The multi-tyrosine kinase inhibitor (TKI) sorafenib is the first drug to be granted approval by U.S. Food and Drug Administration (FDA) for treating advanced HCC. Recently, other TKIs like lenvatinib commonly used in first-line treatment, and regorafenib, cabozantinib, and ramucirumab used in second-line treatment are also approved because of survival advantages in phase III clinical trials^3^. With the development of immunotherapy, immune checkpoint inhibitors against PD-1 and CTLA-4 have also been approved by the FDA for the second-line treatment of HCC^4^. However, the limited median survivals of these patients after treatment with 12–13 months using first-line treatments and 9–11 months using second-line treatments are still an outstanding issue^5^. Identifying more feasible targets and using predictive biomarkers to screen suitable patients is therefore necessary. Furthermore, comprehending mechanisms underlying drug response or resistance and exploring combinational therapies are imperative to improve the efficacies of HCC immunotherapies and the patients’ survival.

A tumor is a type of organoid structure consisting of malignant cells and their environment. The occurrence and development of liver tumors have been attributed to altered signaling pathways and tumor microenvironments (TMEs) involving vasculature, inflammatory cells, stromal cells, and the extracellular matrix (ECM)^6^. Therefore, effective treatment strategies need to understand both factors.

The Wnt/β-catenin signaling pathway is extremely conserved and tightly regulated. It plays a vital role in liver biology. Despite inactivity in most mature and healthy livers, this signaling pathway can be reactivated during cell renewal and/or regeneration processes, some pathological conditions, also diseases and cancer^7^. Abnormal Wnt/β-catenin signaling induces the occurrence and/or progress of various liver diseases, including cancer. In the 2 most common types of primary liver cancer, HCC and cholangiocarcinoma, the Wnt/β-catenin signaling is often hyperactivated and promotes the initiation and progression of tumors^8^. Activating mutations of *CTNNB1*, which codes β-catenin, have been detected in 11%–37% of HCC patients, and inactivating mutations of *Axin1* or *APC* exist in approximately 5%–15% or 1%–2% of liver cancer specimens, respectively, acting as negative regulators^9^.

In this review, we describe the complex interactions involving the Wnt/β-catenin signaling pathway in the TME of HCCs. We also describe the clinically targeted therapies currently designed for this pathway as well as preclinical and clinical applications encompassing Wnt/β-catenin signaling in HCC treatments with sorafenib or immune checkpoint inhibitors. Understanding the role of the Wnt/β-catenin signaling pathway in TME can help us understand the initiation and progression of liver cancer, and provide new avenues for targeted treatment of this disorder.

## The Wnt/β-catenin signaling pathway

The Wnt signaling pathway participates in many basic processes of embryonic development and homeostasis. It is commonly classified into canonical or Wnt/β-catenin signaling pathways and non-canonical pathways, including planar cell polarity and the Wnt/calcium signaling pathway^10^.

Wnts belong to a highly evolutionally conserved, secreted protein family, which is necessary for triggering the Wnt signaling pathway. The human Wnt family containing 19 gene-encoding cysteine-rich glycoproteins bind to more than 15 receptors and co-receptors^11,12^. The Wnt pathway has been shown to participate in cell proliferation, survival, self-renewal or differentiation, and tissue patterning of multicellular organisms^13^.

**Figure 1:**
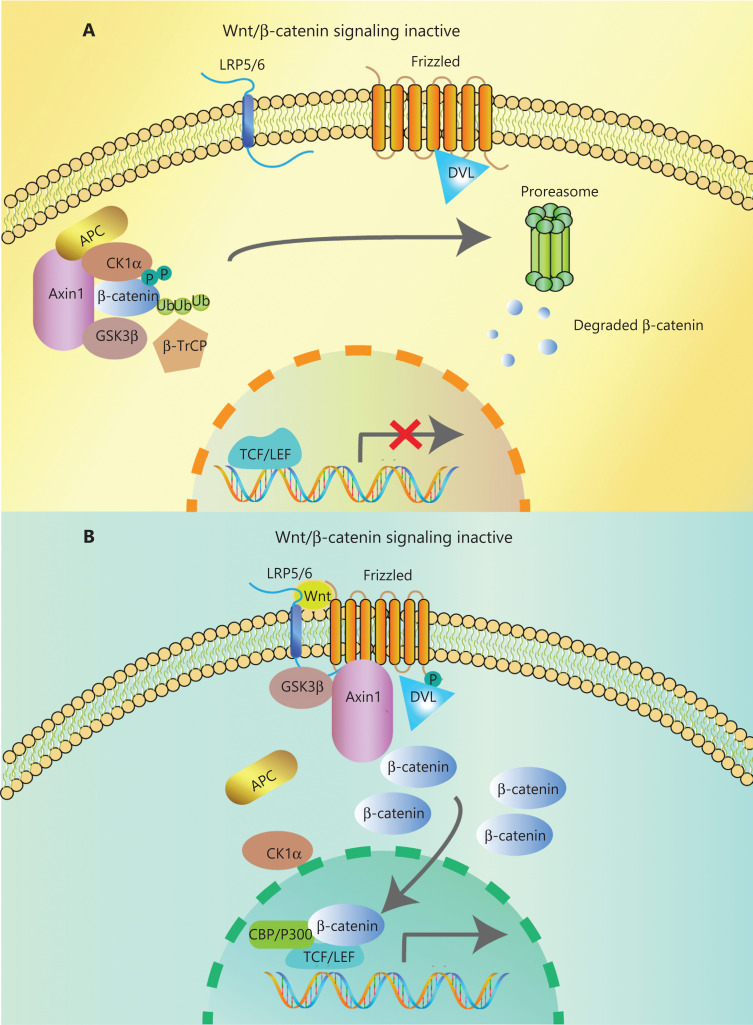
Overview of Wnt/β-catenin signaling. (A) In the absence of Wnt ligands, a degradation complex composed of the kinases, GSK3β, and CK1α, and the scaffolding proteins APC and Axin, is formed. Phosphorylated β-catenin is targeted for proteasomal degradation after ubiquitination by β-TrCP. (B) Wnt ligands bind to the Frizzled/Lrp5/6 receptors, leading to the phosphorylation of Dvl. Dvl recruits Axin and GSK3β, inhibiting the interaction with other components of the degradation complex. Then, β-catenin accumulates in the cytoplasm and translocates to the nucleus, where it interacts with transcription factors of the TCF/LEF family and co-activators such as CBP and P300, promoting the transcription of Wnt target genes.

In the canonical pathway, the Wnt ligands (Wnt1, Wnt2, Wnt3, Wnt3a, Wnt7a, Wnt7b, Wnt8a, Wnt8b, Wnt10b, or Wnt16) bind to corresponding Frizzled receptors (10 kinds found in human and mice) and low-density lipoprotein receptor-related proteins 5/6 (LRP5/6) co-receptors on the cell surface, to initiate intracellular signal transduction through nuclear translocation of β-catenin^14^. The β-catenin is composed of 3 domains: the N-terminal region containing the phosphorylation sites for CK1α and GSK-3β, the armadillo repeat including 12 copies, and the C-terminal domain interacting with TCF/LEF. When Wnt ligands are absent, most β-catenin combines with E-cadherin in adherence junctions at the plasma membrane. The β-catenin in the cytoplasm is degraded by a degradation complex consisting of adenomatous polyposis coli protein (APC), the Axin scaffolding protein, and 2 kinases, casein kinase I isoform-α (CK1α) and glycogen synthase kinase 3β (GSK-3β)^15^. CK1α and GSK-3β can separately phosphorylate Ser45 and Thr41, Ser37, and Ser33 at the N-terminus of β-catenin. Then, β-transducin repeat-containing protein (β-TrCP), a component of the E3 ubiquitin ligase complex, recognizes phosphorylated β-catenin. Finally, β-catenin is polyubiquitinated and undergoes proteasome degradation^16^ (**Figure 1A**). However, when the Wnt ligand binds to the frizzled protein receptor cooperative with LRP5/6, phosphorylation of Dishevelled protein (DVL) and recruitment of Axin and other degradation complex components are induced, leading to dissociation of the degradation complex, inhibition of β-catenin phosphorylation, and nuclear transportation^17^. Axin proteins are rapidly degraded by tankyrase enzymes *via* ubiquitin-mediated proteasomal degradation. In the nucleus, β-catenin acts with the transcription factors of T cell Factor/Lymphoid Enhancer Factor (TCF/LEF) family. P300, CREB-binding protein (CBP), Pygo, BCL9, and other transcriptional co-activators are recruited for initiating transcription of Wnt target genes mediating the formation of the extracellular matrix, anti-apoptosis, cell proliferation (*CJUN*, *MYC*, and *CYD1*) and angiogenesis (*VEGF* and *c-MET*)^18,19^ (**Figure 1B**). Mutations of key genes in this process can prevent β-catenin phosphorylation and then abrogate its degradation. The consequence of increasing β-catenin protein levels in both cytoplasm and nucleus results in the constitutive activation of Wnt/β-catenin pathway.

## The Wnt/β-catenin signaling pathway in the TME

### Wnt-mediated shaping of tumor immunity

**Figure 2:**
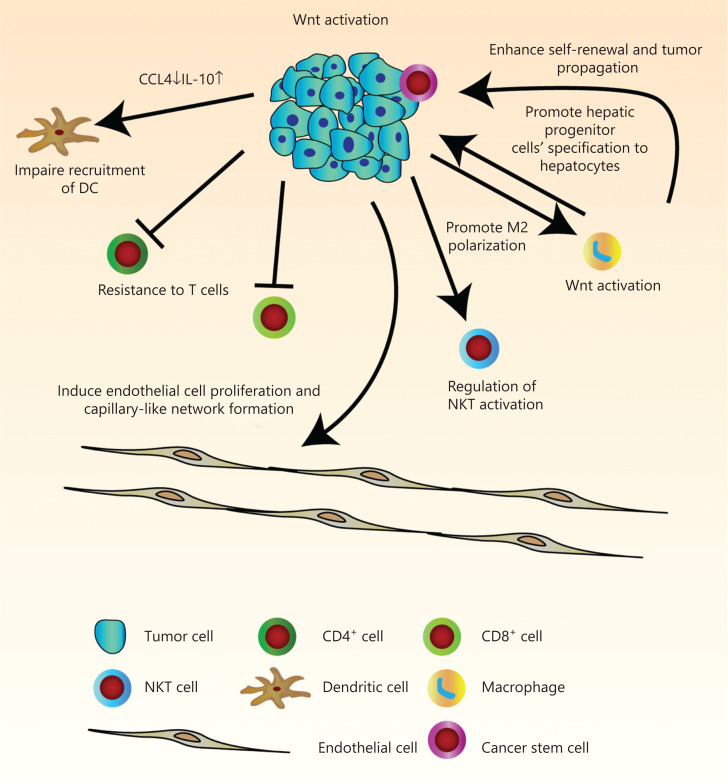
Pleiotropic roles of Wnt/β-catenin signaling in tumor immunity in hepatocellular carcinoma (HCC). Wnt signaling regulates many features of components in the tumor microenvironment. Activation of Wnt signaling in HCC cells can induce endothelial cell proliferation and capillary-like network formation through regulation of angiogenic factors. Due to decreased levels of chemokines, CCL4 and IL-10, recruitment of dendritic cells is defective. Endogenous β-catenin activation of tumor cells can also result in resistance to effector/memory T cells and regulation of liver NKT cell activation. The hepatic tumor cells stimulate M2 macrophage polarization by activation of the canonical Wnt/β-catenin signaling pathway. In turn, Wnt ligands secreted from macrophage can activate the Wnt signaling pathway in tumor cells to promote malignancy, and in hepatic progenitor cells to promote specification to hepatocytes. It can also drive protumoral activation and cancer stem cell self-renewal.

The immune infiltrate refers to the entire encompassing immune cells in the TME. They have been thought to be involved in the regulation of tumor development. There is a great need for more comprehensive knowledge of tumor immunity. We have therefore summarized current findings of the manipulation of tumor infiltrates by Wnt/β-catenin signaling, which has allowed us to better understand multi-component antitumor responses to guide us in the development of more effective immunotherapies for HCC patients (**Figure 2** and **Table 1**).

**Table 1:** The roles of activating Wnt/β-catenin signaling upon immune infiltrating cells in hepatocellular carcinoma

Wnt ligand	Cellular sources	Target cells	Function	Reference
Wnt 3a	Hepa1-6	kclTAMs	Stimulate proliferation of kclTAMs and facilitate tumor growth and hepatic metastasis	^26^
Wnt 3a	Hepa1-6	Macrophage	Promote M2 macrophage polarization, result in tumor growth, migration, metastasis	^25^
Wnt 7a and 10a	Macrophage	Tumor initiating cells	Promote growth of tumor progenitor cells and increase risk of steatosis-induced tumorigenesis	^23^
–	–	HCC tumor cells	Abort DC recruitment, blunt T cell activation, related to increased Treg cells and decreased NK cells and B cells	^33,34^
Wnt 1	Myeloid cells	NKT cells	Regulate IFN-γ responses	^45^

#### Macrophages

Tumor-associated macrophages (TAMs) exert their effects on the development of inflammation-related cancers, including HCC, as the main components of the TME. Macrophages can be polarized into M1 macrophages, which have anti-tumor functions induced by IFN-γ alone or with lipopolysaccharides and M2 macrophages, which exhibit pro-tumor function induced by IL-4 and IL-13^20^. There is emerging evidence showing that TAMs aggravate tumor development, vascularization, metastasis, and suppress acquired immunity by producing various cytokines, chemokines, growth factors, and matrix metalloproteinases (MMP)^21^. It has been reported that Wnt ligands from macrophages can activate the Wnt signal pathway in oncocytes. In chronic liver disease, after phagocytosis of hepatocyte debris, hepatic macrophages secrete Wnt-3a, as an activator of Wnt/β-catenin signaling in hepatic progenitor cells (HPCs) to promote their differentiation into hepatocytes^22^ (**Figure 2**). Macrophages are crucial sources of Wnts in the liver during steatosis. Steatosis-induced activation of Wnt/β-catenin promotes the propagation of tumor initiating cells and progression of tumorigenesis^23^ (**Figure 2**). One study reported that liver regeneration was initiated through β-catenin activation in hepatocytes after partial hepatectomy. This kind of β-catenin activation is regulated by Wnt secreted from endothelial cells and Kupffer cells^24^. Moreover, Wnt/β-catenin can also be triggered inside macrophages by secreted Wnt ligands from cancer cells. The hepatic tumor cells can activate the Wnt/β-catenin signaling pathway by a paracrine function to stimulate M2 macrophage polarization, which promotes protumor activity^25^. In the tumor mouse model, orthotopically inoculated hepatic Hepa1-6 and Wnt3a secreted from Hepa1-6 can activate Wnt/β-catenin signaling and also function as a mitogen for Kupffer cells like TAMs (kclTAMs). However, whether there are other secreting Wnt ligands or mitogens playing roles in the modulation of kclTAM propagation needs further investigation^26^. It was also verified that one kind of long noncoding RNA, LINC00662, could activate the Wnt/β-catenin pathway in an autocrine manner by increasing Wnt3a expression and secretion, and also in a paracrine manner to promote M2 macrophage polarization, leading to HCC tumorigenesis and metastasis *in vivo*^27^ (**Figure 2**). Therefore, in future work, efficient strategies for HCC treatment involve inhibiting secretion of Wnts from hepatoma cells or macrophages, as well as inhibiting activation of Wnt/β-catenin signaling.

#### Dendritic cells (DCs)

DCs function as specialized cells that recognize and present antigens to T cells, with antigenic specificity to modulate T cell functions. DCs are crucial in regulating adaptive immunity. Notch signaling triggers expression of more than 8 Frizzled receptors, to activate the downstream canonical Wnt pathway in hematopoietic progenitor cells, thereby favoring differentiation of DCs^28^. Expressions of β-catenin and GSK-3β are higher in DCs than in monocytes. Crosstalk of the Wnt and MAPK signaling pathways has been implicated in DC differentiation, maturation, and function^29^. It has been reported that tumor-intrinsic constitutive activation of β-catenin can affect antitumor immunity by defective recruitment of DCs into the tumor microenvironment in melanomas^30^, and the failure of DC recruitment is partly due to decreased chemokine CCL4 and high IL-10 production resulting from β-catenin activation within tumor cells^31,32^. In HCC, it was also found that the *CTNNB1* mutation correlated with reduced DCs^33^. The activation of β-catenin promotes immune evasion, which can be ascribed to failing recruitment of DCs, which in turn influences antigen-specific T cell activity^34^.

#### Lymphocytes

The lymphocyte infiltration density of most malignant cancer tissues is heterogenous. Based on the cytotoxic T cell infiltrate within the tumor, it was called a cold or hot tumor in immunotherapy terminology^35^. For many tumors, increased CD8^+^ T lymphocytes have been associated with a better prognosis. Nonetheless, the genetic and epigenetic mechanisms underlying the differences of T cell infiltration among tumors are not clear. For HCC, about one-third of patients with gain-of-function mutations of *CTNNB1* are relevant to scarce intratumoral T cell content^36^. HCC patients with *CTNNB1* mutations have significantly reduced CD8^+^, CD4^+^ T cells, Th2 cells, Tfh cells, and B cells^33^. Luke et al.^37^ concluded by analyzing solid tumors in TCGA that tissue inflammation lacking T cells occurred frequently in primary cancers including HCC, and they found a correlation between tumor intrinsic activation of Wnt/β-catenin and non-T cell-inflamed TMEs. Mei et al.^38^ revealed that DVL1 expression and the infiltration levels of CD4^+^ T, CD8^+^ T, and B cells in HCC were negatively correlated. Blockade of Wnt signaling activation in HCC-bearing mice increased CD4^+^ and CD8^+^ T cells^39^. By employing a novel HCC mouse model with the hydrodynamic tail vein delivery of exogenous antigens, and the CRISPR technique aiming at antioncogenes and genetic elements encoding oncogenes, it has been functionally demonstrated that β-catenin activation results in immune escape and tolerance to anti-PD-1 therapy. This can be partially interpreted by abortively producing chemokine CCL5, which affects the supplementation of CD103^+^ DCs, further influencing antigen-specific CD8^+^ T cells in the liver, leading to decreased immune surveillance^34^. Treg cells, as important immunosuppressive cells, have been proven to be regulated by β-catenin through activation of CCL28 in gastric cancer^40^. Inhibition of the β-catenin/BCL9 interaction can suppress Treg cell infiltration through the TGF-β-CCL20/CCL22 axis in colorectal cancer^41^. In HCC-bearing mice, inhibition of Wnt activation lead to reduced CD25^+^Foxp3^+^ Treg cells^39^. Based on these observations, we speculate that there is a connection between Wnt signaling and Treg cells. However, whether and how Wnt/β-catenin signaling directly regulates Treg cells remains unclear in studies of HCC.

Natural killer T (NKT) cells are important innate-like hepatic lymphocytes implicated in immune responses during infection, cancer, and autoimmunity^42^. NKT cells are divided into 2 major subsets having opposing roles in immune responses by secreting proinflammatory or antiinflammatory chemokines and cytokines^43^. Invariant NKT cells and the chemokine-like factor, LECT2, co-regulate the progression of tumors at the cellular and molecular levels, respectively, in β-catenin-induced HCCs^44^. A study using mouse model also revealed the complex functions of Wnt/β-catenin signaling in regulating hepatic NKT cell activation. The imposing effects of β-catenin on NKTs are dependent or independent on Wnt^45^.

### Wnt/β-catenin in cancer stem cells (CSCs)

CSCs refer to a subpopulation of poorly differentiated cells that are qualified with the same self-renewing and pluripotent potentials as common somatic stem cells (SSCs)^46,47^. There is emerging evidence that CSCs within the TME are associated with aggressive tumor progression, chemoresistance, and recurrence in HCC patients. Hepa1-6 CSC spheroids with activating β-catenin have a stronger *in vivo* tumorigenic ability in the immunocompetent liver microenvironment of a HCC orthotopic mouse model^48^. EPCAM, LGR5, CD44, and CD133 are representative cell surface markers of CSCs^49–51^. A subset of “superpotent CSCs”-Wnt-activity^high^ALDH1^+^EpCAM^+^ triple-positive cells with the highest tumorigenesis capacity among all HCC cells promotes Wnt/β-catenin signaling by increased DVL1^52^. HCC is believed to originate from CSCs. The existence of autocrine Wnt signaling, particularly Wnt3 in EpCAM^+^ CSCs from HCC, was reported in 2017^53^. *LGR5* is the target gene of the canonical Wnt signaling. In LGR5^+^ circulating stem/progenitor cells, Wnt, and R-spondin receptor encoded by LGR5 further upregulate the expressions of CD44 and CD133^54^. Wnt signaling can also be activated in liver CSC by a long noncoding RNA called lncTCF7, which attracts the SWI/SNF compound to the TCF7 promoter, thus inducing CSC self-renewal and tumor propagation^55^. Besides, Fan et al.^56^ discovered that CSC-like traits, tumorigenicity, and sorafenib-resistance in HCC were enhanced by active Wnt/β-catenin pathway stimulated by the nonreceptor protein tyrosine kinase 2. Activation of the Wnt/β-catenin signaling pathway induced by Ring1 also promotes transformation of HPCs into liver cancer stem-like cells^57^. In addition, β-catenin is involved in Shp2-promoted liver CSC expansion connected with patient responses to chemotherapeutics^58^. In mouse hepatic stellate cells and liver tumor-initiating stem cell-like cells, the Wnt/β-catenin pathway regulates stearoyl-CoA desaturase expression to stabilize *LRP5* and *LRP6* mRNA, further augmenting Wnt signaling to result in liver fibrosis and tumor development^59^. The epithelial-mesenchymal transition (EMT) has been confirmed to be closely related to activation of CSC in tumor tissues^60–62^. It has also been suggested that the EMT is regulated by the Wnt/β-catenin pathway in HCC^63,64^.

Understanding the pathways that regulate CSC self-renewal, differentiation, and tumorigenicity are important for the development of anti-cancer therapies. Conserved signaling pathways like Notch, Hedgehog, and Wnt pathways play important roles in the function of normal stem cells and are also implicated in cancer^65^. Small molecular inhibitors targeting these associated pathways have been implicated in preclinical and clinical studies^65–67^. However, the specific mechanism of how the Wnt signaling pathway orchestrates interactions between CSCs and the TME needs further investigation.

### The Wnt-mediated construction of tumor angiogenesis

Tumor angiogenesis is a key factor in maintaining tumor growth. Angiogenic factors take part in tumor angiogenesis, infiltration, and metastasis. At the molecular level, angiogenesis is a consequence of competition between proangiogenic factors and inhibitors. Many key angiogenic factors have been discovered, including vascular endothelial growth factor, matrix metalloproteinase, fibroblast growth factor (FGF), platelet-derived growth factors, angiopoietins, hepatocyte growth factor, and hypoxia-inducible factor^68^. HCC has properties of hypervascularization and vascular abnormalities. Tumor vascularity can expand by enrolling existing vessels into the tumor zone in HCC^69^. The Wnt/β-catenin signaling pathway regulates angiogenic factors. Enhanced Wnt/β-catenin-mediated FGF18 expression and secretion are induced by ribosomal protein, S15a, which promotes angiogenesis and tumor growth in HCC cells^70^. Yang et al.^71^ reported that miR-1301 restrained HCC cell transplantation, aggression, and vascularization by reducing Wnt/β-catenin signaling by targeting BCL9, a coactivator of β-catenin transcription. Endotheliocytes express many Wnt pathway-associated elements, and the activation of Wnt1 can induce endotheliocytes expansion and capillary-like network arrangements (**Figure 2**)^47,72^. Functional studies of HCC by Hu et al.^73^ also showed that Wnt signaling was necessary for angiogenesis. Wnt antagonists inhibit tube formation and decrease microvessel density and angiogenic factors. The study clarified the antitumor potential of Wnt antagonists in increasing cell apoptosis and in attenuating tumor angiogenesis.

### Wnt signaling and noncellular components

In addition to various cell types, the TME also includes noncellular components involving the ECM, growth factors, proteolytic enzymes, and their inhibitors^74^. The ECM participates in the initiation and progression of tumors, and also affects cancer therapy^75^. Cysteine-rich 61, a member of the CCN growth factor family and also an ECM protein, is a target gene of β-catenin and promotes the progression of HCC through regulation of hepatic stellate cells^76,77^. Dermatopontin is an ECM protein implicated in intercellular adhesion and ECM development. Liu et al.^78^ reported that dermatopontin repressed HCC proliferation by inactivating pro-oncogenic Wnt/β-catenin signaling through inhibition of CXX4. As for the growth factors in the TME, the intricate crosstalk of Wnt/β-catenin and the epidermal growth factor receptor, FGF pathways have been discussed in the context of HCC^79,80^. Upregulation of FGF15/19 signaling activates canonical Wnt signaling and the EMT to facilitate HCC in a metabolic disorder background^81^. Overexpression of platelet-derived growth factor B enhances expression of pro-oncogenic factors, including β-catenin, VEGF, and platelet endothelial cell adhesion molecule-1^82^. MMPs are a family of endoproteases, which degrade the ECM including collagen, gelatin, and elastin. It is thought that MMP-2 and MMP-9 are direct targets of Wnt/β-catenin signaling^83^. Activation of the Wnt/β-catenin pathway promotes the EMT, migration, and malignant development of HCC by regulating MMP^84,85^. Exosomes are small extracellular vesicles ranging in size from 30 to 150 nm, and are increasingly regarded as important carriers of information in the TME^86^. It has been shown that exosomes contributed to HCC development and progression^87,88^. Yu et al.^89^ showed that hypoxia-induced exosomes activated the Wnt/β-catenin pathway and promoted proliferation, migration, and the EMT in normoxic HCC cells.

### Crosstalk with other pathways in the TME

The interaction between the Wnt and Notch signaling pathways participates in many developmental processes. Cells can use the pattern Notch-ON/Wnt-OFF or Wnt-ON/Notch-OFF to regulate signaling. These 2 pathways modulate each other at different signal transduction levels^90^. A study reported that in liver cancer stem cells, Notch negatively regulated active β-catenin protein expression, and Notch1 was downstream of Wnt/β-catenin signaling^91^. In mice KclTAM, activation of Notch signaling impeded *CTNNB1* mRNA. This negative correlation was also confirmed in CD68^+^ macrophages of patient-derived HCC biopsies^92^.

Hippo, Notch, and Wnt/β-catenin signaling interact to maintain liver size and inhibit HCC. The positive interaction of Notch signaling and Hippo signaling effector YAP/TAZ promotes severe hepatomegaly and accelerates initiation and development of HCC. Unexpectedly, canonical Wnt signaling activation inhibits the interaction between YAP/TAZ and Notch signaling. The positive feedback loop was repressed and thus inhibited the formation of HCC^93^. Canonical Wnt signaling activation induces KIF2C expression, then enhances mTORC1 signaling and HCC development by KIFC2 binding to TBC1D7^94^. In HepG2 hepatoma cells, Wnt/β-catenin activation and Snail expression are dependent on ERK1/2 signaling to regulate the EMT^95^. TGFβ signaling also induces Wnt and Sonic hedgehog signaling activation in development of HCC EMT^96^. Zhang et al.^97^ discovered that β-catenin interacted with hif-1α to promote hif-1α signaling and to suppress Wnt/β-catenin signaling, together leading to an exaggerated EMT in hypoxic HCC. These findings further addressed the complexity of the signaling pathway interaction network.

## Therapeutic inhibition of Wnt signaling in the TME

Because of the essential and multiple roles of canonical Wnt signaling in the TME, novel molecular therapies targeting this pathway have shown unlimited prospects. Therapeutic drugs that directly target the Wnt signaling cascade are currently divided into the following categories: i) inhibiting secretion of Wnt ligands using porcupine (PORCN) inhibitors. PORCN is a membrane-bound O-acyltransferase acylating Wnt ligands during the posttranslational modification stage^98^; ii) targeting Wnt signaling by extracellular components like Frizzled and LRP5/LRP6; iii) aiming at cytoplasmic molecules of signaling such as tankyrase and CK1α; and iv) impeding the intranuclear Wnt signaling, including β-catenin and the co-factors involved in the transcription of Wnt target genes^99^. However, there is currently no approved therapeutic agent for clinical use that can specifically and effectively target this pathway (**Table 2**). One of the major concerns is that inhibition of the Wnt signal pathway may affect Wnt-dependent stem cells and the regeneration of tissues and organs. Some modulators targeting different key steps of the Wnt/β-catenin pathway have been evaluated for their depressor effect and safety in clinical trials^100^. Among these trials, one study aiming at hepatocellular carcinoma using a humanized monoclonal antibody against Dickkopf-1 (DKK1), DKN-01, is involved in phase I and II trials (ClinicalTrials.gov Identifier: NCT03645980). DKK1 is a secreted glycoprotein inhibiting the canonical Wnt pathway, by binding to the LRP5/6 co-receptor. The role of DKK1 is controversial. Qin et al.^101^ found inhibition of DKK1 expression facilitated β-catenin to translocate into nuclei, which may influence the proliferation and metastasis of HCC cells. Contrary to the hypothesis that DKK1 is supposed to function as a tumor suppressor, recent studies showed its oncogenic role in activating Wnt/β-catenin signaling pathway, thus promoting the invasion, metastasis, and tumorigenicity of HCC cell lines^102,103^. DKK1 has been shown to have protumor effects and was correlated with poor prognosis, rendering DKK1 an attractive anticancer target^104^. Moreover, Liang et al.^105^ injected tumorigenic β-catenin and MET into β-catenin-deficient livers to induce cancer, and unexpectedly found that Sox9^+^ cells with stem cell properties expanded inflammatory, upregulated cytokines, and several pro-tumorigenic pathways such as Akt, Erk, and Wnt/β-catenin were also enhanced to exacerbate HCC progression. Restraining β-catenin in the liver startingly creates a pro-tumorigenic microenvironment to aggravate HCC driven by β-catenin/MET, further adding to the complicacy of this pathway. It is also worth noting that as traditional Chinese medicines have gained constant attention because of potential anticancer abilities, several of them have been used in the treatment of HCC by targeting the immunosuppressive TME^106^. Some traditional Chinese medicines were found to suppress proliferation, induce apoptosis, and impair invasion of liver cancer cells by blocking the Wnt/β-catenin signaling pathway, such as agkihpin (a snake venom arginine esterase)^107^, *Dendrobium candidum* extract^108^, ethyl acetate extract from Jiedu Xiaozheng Yin^109^, and *Zanthoxylum avicennae*^110^. Further animal studies and clinical trials are warranted.

**Table 2:** Wnt/β-catenin signaling inhibitors in current clinical trials

Drug	Target	Cancer type	Phase	Clinicaltrials. Govidentifier
LGK974	PORCN	Melanoma, breast cancer and pancreatic CA	I	NCT01351103
ETC-1922159	PORCN	Solid tumors	I	NCT02521844
CGX1321	PORCN	Colorectal adenocarcinoma Gastric adenocarcinoma Pancreatic adenocarcinoma Bile duct carcinoma Hepatocellular carcinoma Esophageal carcinoma Gastrointestinal cancer	I	NCT03507998
DKN-01	DKK1	Hepatocellular carcinoma	I/II	NCT03645980
Niclosamide	Axin1	Metachronous or synchronous metastases of a colorectal cancer progressing after therapy	II	NCT02519582
PRI-724	β-Catenin	Advanced pancreatic cancer Metastatic pancreatic cancer Pancreatic adenocarcinoma	I	NCT01764477
OMP-18R5 (Vantictumab)	Frizzled receptor	Breast cancer	Ib	NCT01973309
OMP-54F28 (Ipafricept)	Frizzled family receptor 8	Solid tumors	I	NCT01608867
SM08502	Wnt pathway related-gene expression	Solid tumors	I	NCT03355066

## Preclinical and clinical applications involving the Wnt/β-catenin pathway

Sorafenib is the standard therapy for patients with advanced HCC. Sorafenib has been shown to suppress Wnt/β-catenin signaling^111^. In turn, β-catenin can diminish apoptosis and cell growth inhibition induced by sorafenib treatment. Up-regulation of β-catenin contributes to HCC resistance^112^. Lin et al.^113^ confirmed that suppression of canonical Wnt signaling could improve the therapeutic effect of sorafenib alone in a HCC cell line and mouse xenograft model. In a preclinical pediatric HCC model, inhibition of β-catenin activity using the ICG001inhibitor combined with sorafenib led to lower cell viability and showed synergistic effects, indicating that blockade of Wnt/β-catenin signaling may attenuate sorafenib resistance^114^. Combinatory treatment using FH535, an artificial inhibitor of Wnt/β-catenin signaling with sorafenib on HCC cells, significantly decreased the autophagic flux and increased apoptosis^115^. Immunotherapy with checkpoint inhibitors is also an important emerging treatment option for HCC. Nivolumab in first-line trials and pembrolizumab in second-line trials showed promise of efficacy, but neither reached statistical significance^116^. Clinical trials have shown that only about 15%–20% of HCC patients showed an objective response to these therapies. Harding et al.^117^ found that activating mutations of the Wnt pathway partly contributed to innate resistance to immune checkpoint blockade, and clinical findings displayed shorter median progression-free survival and median overall survival. Immune exclusion HCC class, characterized by mutations in the Wnt/β-catenin, are refractory to immune checkpoint blockers^5^. Du et al.^118^ found that EGFR activation and a Wnt ligand up-regulated PD-L1 expression by binding the β-catenin/TCF/LEF transcriptional complex to the *CD274* gene promoter in glioblastoma immune evasion. Consistent with this finding, Deng et al.^119^ identified β-catenin as a transcriptional factor for PD-L1. An innate immune effector, ISG12a, inhibits Wnt/β-catenin signaling by inhibiting proteasomal degradation of Axin, thereby repressing immune checkpoint PD-L1 expression and sensitizing cancer cells to NK cell-mediated killing in HCC and gastric cancer. There are 20%–30% of advanced HCC patients carrying gain-of-function mutations in β-catenin, which is the third most frequent mutation following the hTERT promoter and TP53. This group of patients is unlikely to benefit from immune checkpoint inhibitors^34^. However, it is promising to utilize alterations in the β-catenin pathway as a biomarker to direct therapy for HCC patients. Checkmate459, a randomized phase III clinical test comparing nivolumab and sorafenib in first-line HCC therapy, failed to meet its primary endpoint, which clearly emphasized the urgent need for patient selection^36^. Detailed studies of marker candidates can be used to stratify clinical trials and improve guidance for therapeutic decisions for HCC patients.

## Conclusions and perspectives

This review highlights the roles of the Wnt/β-catenin pathway in the TME of HCC, including its complicated roles in immune infiltration, cancer stem cell tumor vascularization, and its interaction with noncellular components in the TME. What increases the difficulty of studying this pathway is that the Wnt/β-catenin pathway also crosstalks with other pathways like Notch, Hedgehog, and Hippo. Furthermore, because of the tumor heterogeneity in HCC, it is reasonable that the diversified activation of the Wnt pathway could exist in tumor nodules, which results in more intricate Wnt signaling in response to variant stress, TKI treatment, and immunotherapy. These heterogeneities may lead to tumor resistance and treatment failure. Thus, designing specific targeted drugs and individualized therapies for different patients according to their tumor molecular typing and risk factor is warranted.

Tumors with mutations in the Wnt/β-catenin pathway are characterized by immune-exclusion in the TME. Both the activation and inactivation mutations of *CTNNB1* are involved in liver tumor development. Precise modulation of Wnt/β-catenin is therefore essential to equilibrize anticancer effectiveness and adverse events, to address the challenges for advancing and prospective clinical tests. In spite of these fears, new modulators of the Wnt/β-catenin signaling provide us with opportunities to enhance our understanding of this extremely complex pathway, and may be used to treat Wnt-related diseases including tumors. Although several traditional Chinese medicines have been shown to inhibit Wnt/β-catenin signaling in HCC cells, relevant clinical trials and data are still lacking. The efficacy of most Chinese herbs is often dependent on a variety of components directed to multiple targets, including altering the TME and the systemic immune system. It is therefore challenging to develop a single active ingredient from traditional Chinese medicine. Considering that Wnt/β-catenin mutations exert effects on the therapy of HCC patients, including sorafenib and immune checkpoint inhibitors, they can be used as biomarkers to predict resistance to therapies and applications of Wnt modulators in combination with immunotherapy regimens for the development of novel cancer treatments. It is also worth using traditional Chinese medicine combined with immunotherapy to treat HCC, in view of their inhibitory effects on Wnt signaling. The application of Wnt modulators in preclinical and clinical studies remains to be corroborated and deserves further exploration in advanced HCC. A precise and detailed understanding of the contributions of Wnt/β-catenin signaling to diverse compositions in the TME in HCC is a prerequisite to thoroughly comprehend data from preclinical and clinical studies, to provide a foundation for conceiving better therapeutic regimens targeting Wnt/β-catenin signaling in HCC. It is promising that the emerging single-cell-based omics technology, including but not limited to scRNA-seq, scATAC-seq, single-cell mass cytometry, and spatial transcriptome analysis, have enabled microscopic interpretation of cellular behaviors and immune mechanisms. These new techniques have helped us to understand tumor heterogeneity, immune characteristics of tumors, the TME, tumor recurrence, and cancer treatment more comprehensively.
